# Clinicopathologic and endoscopic features of early-stage colorectal serrated adenocarcinoma

**DOI:** 10.1186/s12876-017-0702-x

**Published:** 2017-12-12

**Authors:** Daiki Hirano, Shiro Oka, Shinji Tanaka, Kyoku Sumimoto, Yuki Ninomiya, Yuzuru Tamaru, Kenjiro Shigita, Nana Hayashi, Yuji Urabe, Yasuhiko Kitadai, Fumio Shimamoto, Koji Arihiro, Kazuaki Chayama

**Affiliations:** 1 Department of Gastroenterology and Metabolism, Hiroshima University Hospital, 1-2-3 Kasumi, Minami-ku, Hiroshima, 734-8551 Japan; 2 Department of Endoscopy, Hiroshima University Hospital, Hiroshima, Japan; 3 Department of Anatomical Pathology, Hiroshima University Hospital, Hiroshima, Japan; 4 Department of the Faculty of Human Culture and Science, Prefectural University of Hiroshima, Hiroshima, Japan; 5 The Faculty of Humanities and Human Sciences, Hiroshima Shudo University Hiroshima, Hiroshima, Japan

**Keywords:** Serrated adenocarcinoma, Colorectal cancer, Narrow band imaging, Pit pattern

## Abstract

**Background:**

Serrated adenocarcinoma (SAC) is a distinct colorectal carcinoma variant that accounts for approximately 7.5% of all advanced colorectal carcinomas. While its prognosis is worse than conventional carcinoma, its early-stage clinicopathologic features are unclear. We therefore aimed to clarify the clinicopathologic and endoscopic characteristics of early-stage SACs.

**Methods:**

Forty consecutive early-stage SAC patients at Hiroshima University Hospital were enrolled; SACs were classified into epithelial serration (Group A, *n* = 17) and non-epithelial serration (Group B, *n* = 23) groups. Additionally, we classified serrated adenoma into 4 types: sessile serrated adenoma (SSA), traditional serrated adenoma (TSA), unclassified, and non-serrated adenoma type.

**Results:**

There were significant differences between Groups A and B in terms of tumor size (27.6 vs. 43.1 mm), incidences of T1 carcinoma (71% vs. 13%), and having the same color as normal mucosa (47% vs. 17%), respectively (*p* <0.01). In SACs >20 mm, the incidence of T1 carcinoma in Group A (70%) was significantly greater than that in Group B (13%) (*p* <0.05). There were significant differences in ‘Japan NBI Expert Team’ type 3 and type V pit pattern classifications between the 2 groups. The average TSA-type tumor size (42.6 mm) was significantly larger than that of the SSA (17.2 mm) and non-serrated component types (18.3 mm). The incidences of submucosal invasion in SSA- (80%), unclassified- (100%), and non-serrated-type (100%) tumors were significantly higher than that in the TSA type (11%).

**Conclusions:**

Epithelial serration in the cancerous area and a non-TSA background indicated aggressive behavior in early-stage SACs.

## Background

Colorectal carcinoma is one of the most common malignancies in the world. The classical genetic model for colorectal tumorigenesis described by Fearon and Vogelstein is the adenoma-adenocarcinoma sequence, which is driven by the progressive accumulation of critical mutations [1]. In this model, the adenomatous polyp is the principal precursor of colorectal carcinomas [1, 2]. More than 90% of colorectal carcinomas are medullary, micropapillary, mucinous, serrated, or signet ring cell [3]. Serrated adenocarcinomas (SACs) were first described by Jass and Smith [4] and represent the malignant progression of dysplastic serrated lesions, most commonly serrated adenomas. SAC is considered to be one of several end-points of a progression pattern known as the serrated neoplasia pathway [5, 6], which is a major contributor to colorectal carcinoma; approximately 25% of cases arise through this pathway [7, 8]. Such carcinomas originate in serrated polyps such as sessile serrated adenomas (SSAs) and traditional serrated adenomas (TSAs) [9]. SACs arising from SSAs have molecular profiles that are CpG island methylator phenotype-high and *BRAF* mutation-positive, with high microsatellite instability (MSI). SACs arising from TSAs are CpG island methylator phenotype-low, KRAS mutation-positive, and exhibit microsatellite stability or low microsatellite instability [10–12]. Recently, SACs have been described as having less favorable 5-year survival outcomes than conventional colorectal carcinomas [5]. However, there are no reports on the clinicopathologic and endoscopic features in early-stage SACs. Therefore, the aim of this study was to investigate and clarify these features in early-stage SACs.

## Methods

Forty consecutive early-stage colorectal SACs were extracted from 1142 colorectal carcinoma patients (895 with Tis carcinoma and 247 with T1 carcinoma) who were treated at Hiroshima University Hospital between January 2009 and January 2016. Patients with familiar adenomatous polyposis, inflammatory bowel disease, or serrated polyposis syndrome were excluded. The lesions were resected using polypectomy, endoscopic mucosal resection, endoscopic submucosal dissection, or surgical resection.

### Endoscopic examination

Upon detection of a lesion by standard colonoscopy, the surface mucus was washed away with lukewarm water and indigo carmine dye was spread over the lesion. When it was not possible to adequately stain the surface with indigo carmine for diagnosis, crystal violet dye was used instead, and magnifying observation was performed. All images were obtained with magnifying colonoscopies (CF-Q240ZI, CF-H260AZI, and CF-H290ZI; Olympus, Tokyo, Japan) with up to 80-fold magnification in combination with a standard video processor system (EVIS LUCERA system, EVIS EXERA system; Olympus Inc., Tokyo, Japan). Pit pattern diagnosis was based on the dominant pit pattern according to the Kudo and Tsuruta classification [13, 14] as well as the dominant narrow-band imaging (NBI) findings as proposed by the Japan NBI Expert Team (JNET) classification [15, 16]. The JNET classification divides vessel and surface patterns into 4 categories: types 1, 2A, 2B, and 3, which are consistent with the histopathological findings of hyperplastic polyp/sessile serrated polyp, low-grade intramucosal neoplasia, high-grade intramucosal neoplasia/shallow submucosal invasive cancer, and deep submucosal invasive cancer, respectively.

### Pathological examination

Resected specimens were fixed in a 10% buffered formalin solution. Paraffin-embedded samples were then sliced into 2–3-mm sections and stained with hematoxylin and eosin.

The subjects were diagnosed by 2 pathologists (K.A. and F.S.) who were blinded to the endoscopic features of the lesion. Histologic type, depth of tumor, venous invasion, and lymphatic invasion were also categorized according to the Japanese Classification of Colorectal Carcinoma [17]. For submucosal (SM) invasive cancer, we measured the SM depth and budding grade. According to the JSCCR guidelines [18], the method used for measuring the SM depth was as follows: When it was possible to identify or estimate the location of the muscularis mucosae, the depth of SM invasion was measured from the lower border of the muscularis mucosae of the lesion, irrespective of macroscopic type. When it was not possible to identify or estimate the location of the muscularis mucosae, the depth of SM invasion was measured from the surface of the lesion.

Budding is defined as a single cancer cell or a cluster of 5 cells along the invasion margin, and was graded per microscopic field at 200× magnification (i.e., grade 1, 0–4 buds; grade 2, 5–9 buds; and grade 3, ≥10 buds) [19].

Tumors that contained more than one histologic type of carcinoma were classified based on the predominant histologic type. Well-differentiated tubular adenocarcinoma (tub1) was characterized by distinct and large gland formation; moderately differentiated tubular adenocarcinoma (tub2) was composed of medium-to-small glands with a cribriform structure, and poorly differentiated adenocarcinoma (Por) had little tendency to form glands or tubules; however, intracellular mucus production was observed [17].

The current diagnostic criteria for SAC are based on the recognition of a serrated polyp (hyperplastic polyp, SSA, or TSA) next to the carcinoma or of a characteristic carcinoma histology.

The morphological characteristics of SAC were defined using Mäkinen’s criteria, and include epithelial serrations, clear or eosinophilic cytoplasm, abundant cytoplasm, vesicular nuclei, distinct nucleoli, scarceness (<10%) of necrosis, mucin production, and cell balls or papillary rods in the mucin. However, the pathological general definition of SAC is tubular adenocarcinoma with serration. A diagnosis of SAC was considered when the carcinoma met at least 6 of the first 7 features listed above [5] or when the carcinoma was adjacent to a serrated adenoma [20–22]. SACs were diagnosed by 2 pathologists (K.A. and F.S.) and 1 gastroenterologist (D.H.).

### Evaluation

**Fig. 1:**
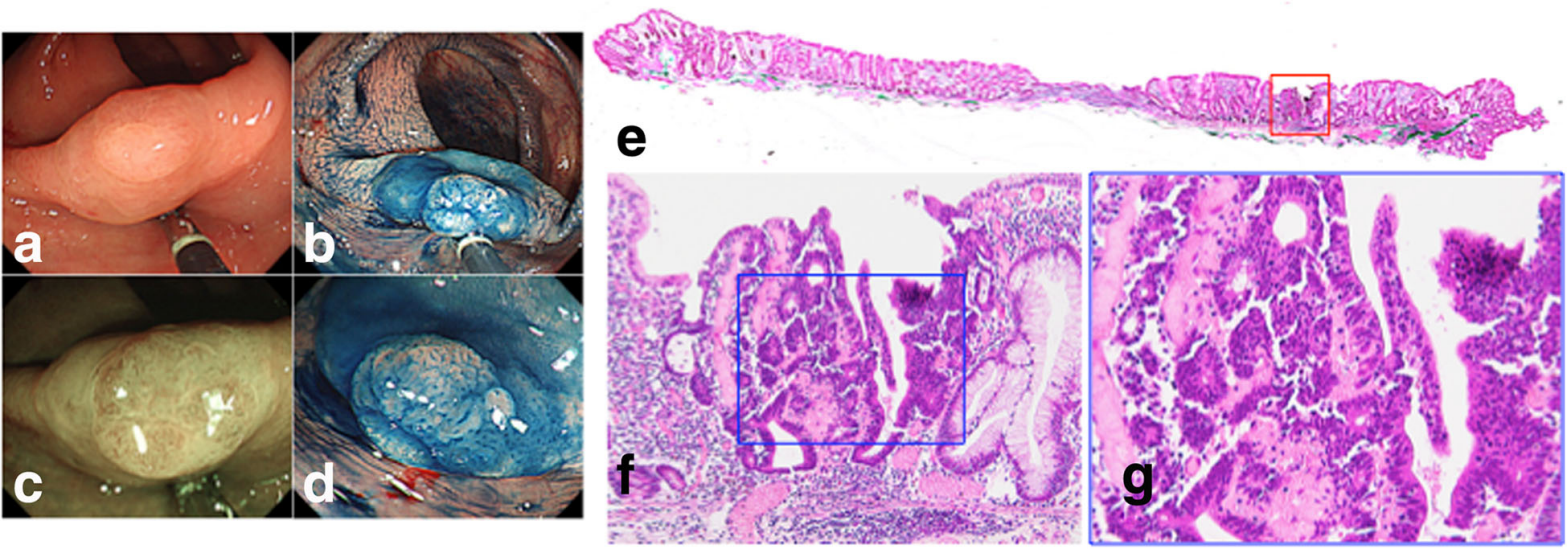
A case of Tis serrated adenocarcinoma with a serrated adenoma. **a** Colonoscopic view of a serrated adenocarcinoma lesion in the ascending colon. **b** Endoscopic findings after indigo carmine spraying; the small elevated nodule in the tumor can be observed. **c** Magnifying narrow-band imaging (NBI) observation. In the tumor lesion, mucosa with a Japan NBI Expert Team classification type B can be observed. **d** Magnifying endoscopic finding after indigo carmine dye spraying. Type II-open-containing normal type II pits are observed in the tumor. **e** Hematoxylin and eosin (HE) staining of the whole specimen. **f, g** High-power view of the HE-stain specimen; a section of adenocarcinoma is shown

**Fig. 2:**
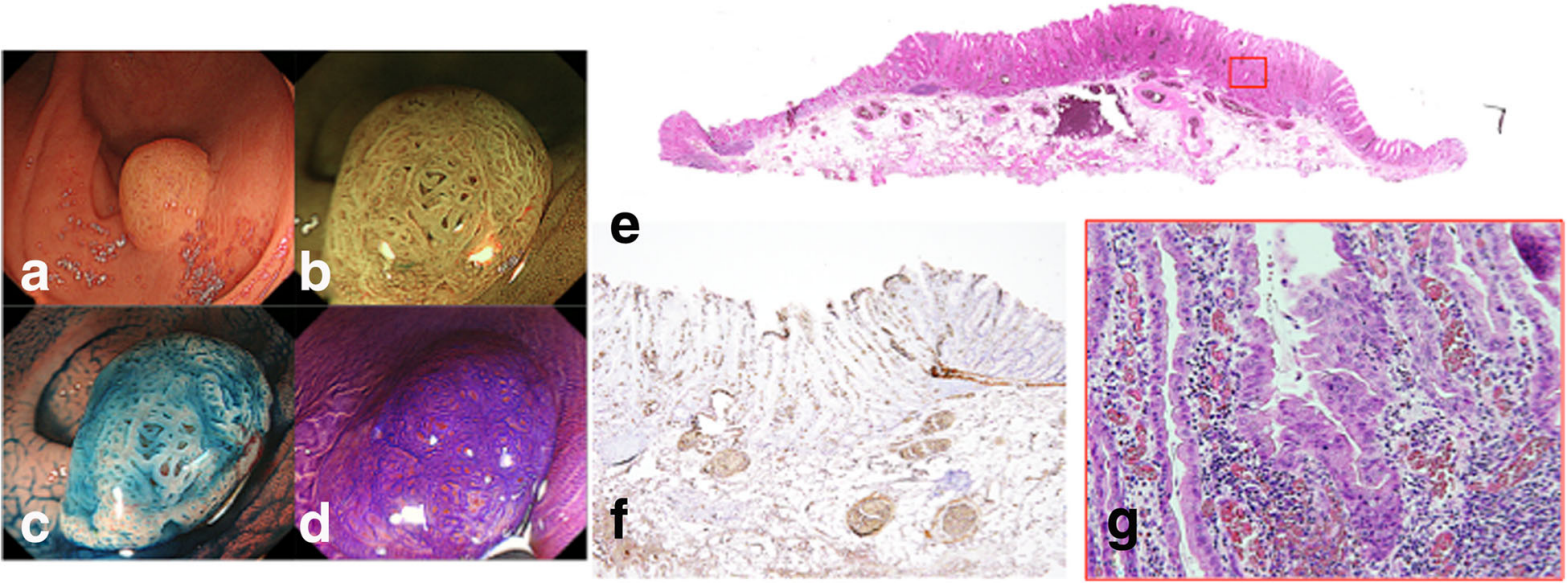
A case of T1 serrated adenocarcinoma without a serrated adenoma. **a** Colonoscopic view of serrated adenocarcinoma in the cecum. **b** Endoscopic view after indigo carmine dye spraying. A 0-Is lesion is clearly delineated. **c** Magnifying narrow-band imaging (NBI) observation; a Japan NBI Expert Team classification type 2B lesion can be observed. **d** Magnifying view of a crystal violet-stained section. **e** Hematoxylin and eosin (HE) staining of the whole specimen. **f** Immunostaining of the specimen with anti-desmin antibody; the muscle fibers are no longer visible. **g** High-power view of HE-stained specimen; epithelial serration is visible, and adenocarcinoma can be observed invading the submucosa

We classified SACs into the 2 groups: those with epithelial serration (Group A, *n* = 17) and those with non-epithelial serration (Group B, *n* = 23); examples are shown in Figs. 1 and 2, respectively. SACs were categorized as having epithelial serration if more than 5% of the cancerous area exhibited such morphology.

We compared the following clinicopathologic characteristics between the 2 groups: sex, age, location, tumor size, and invasion depth (Tis/T1). Lesion location was divided into proximal colon (i.e., proximal to the splenic flexure), distal colon (i.e., distal to the splenic flexure) and rectum. We also compared the following endoscopic findings between the 2 groups: tumor surface color (same as normal mucosa vs. discolored vs. reddish), macroscopic type (protruded vs. superficial), pinecone-like status, varicose microvascular vessels (VMVs) vs. pit pattern, and JNET classification [15, 16]. We also classified the serrated adenoma close to the carcinoma into 4 types: SSA, TSA, unclassified, and non-serrated adenoma type, and compared clinicopathologic and endoscopic features between these 4 types. This study was conducted in accordance with the Declaration of Helsinki and was approved by the Institutional Review Board of our hospital. Written informed consent was obtained from all patients who participated in this study.

### Statistical analysis

Each continuous variable was presented as the mean ± standard deviation. Comparisons of continuous variables were performed using Student’s t-test, and comparisons of dichotomous variables were based on the chi-square and Fisher’s exact tests. JMP version 8 (SAS Institute, Cary, NC) was used to analyze the data. The significance level was set at 5% for each analysis; *p* < 0.05 was considered statistically significant.

This study was conducted in accordance with the Declaration of Helsinki and was approved by the Institutional Review Board of our hospital. Written informed consent was obtained from all patients who participated in this study.

## Results

With respect to clinicopathologic characteristics, there were significant differences in average tumor size, incidence of T1 carcinoma, and incidence of tumor color being the same as normal mucosa between Groups A and B (*p* < 0.01); however, there were no significant differences in sex, age, and tumor location. Furthermore, there were no significant differences in macroscopic type, pinecone-like findings, and VMVs regardless of epithelial serration **(**Tables 1, 2 **)**.

**Table 1:** Characteristics of serrated adenocarcinoma

Variables	Epithelial serration		*p*-value
	Present (*n* = 17)	Absent (*n* = 23)	
Sex (male/female)	9/8	14/9	N.S.
Average age (years)	70.5	68.5	N.S.
Location (proximal colon/distal colon/rectum)	8/1/8	8/1/14	N.S.
Average size (mm)	27.6	43.1	<0.05
Invasion depth (Tis/T1)	5/12	20/3	<0.05

*N.S.* not significant

**Table 2:** Endoscopic features of serrated adenocarcinoma

Variables	Epithelial serration		*p*-value
	Present (*n* = 17)	Absent (*n* = 23)	
Color			
Same as normal mucosa	8 (47)	4 (17)	<0.05
Discolored	1 (6)	4 (17)	N.S.
Reddish	8 (47)	15 (66)	N.S.
Macroscopic type			
Protruded	14 (82)	20 (87)	N.S.
Superficial	3 (18)	3 (13)	N.S.
Pinecone like findings (+)	2 (12)	6 (26)	N.S.
Varicose microvesicular (+)	3 (18)	1 (4)	N.S.

Values in parentheses are percentages (%)

*N.S.* not significant

Endoscopic findings revealed that there were significant differences in the incidence of JNET Type 3 and type V pit pattern between Groups A and B, respectively **(**Tables 3, 4 **)**.

**Table 3:** Narrow band imaging magnification findings in the carcinoma area of serrated adenocarcinoma

Epithelial serration	JNET classification			
	1	2A	2B	3
Present (*n* = 17)	1 (6)	6 (35)	7 (41)	3 (18)^a^
Absent (*n* = 23)	1 (4)	13 (57)	9 (39)	0 (0)^b^
Total (*n* = 40)	2 (5)	19 (48)	16 (40)	3 (7)

a vs. b: *p* < 0.01. Values in parentheses are percentages (%). JNET: Japan NBI [narrow-band imaging] Expert Team

**Table 4:** Pit pattern classification in the carcinoma area of serrated adenocarcinoma

Epithelial serration	Pit pattern				
	II	Open-II	III_L_	IV	V
Present (*n* = 17)	0 (0)	1 (6)	2 (12)	2 (12)^a^	12 (70)^c^
Absent (*n* = 23)	1 (4)	0 (0)	3 (13)	10 (44)^b^	9 (39)^d^
Total (*n* = 40)	1 (3)	1 (3)	5 (13)	12 (30)	21 (53)

a vs. b, c vs. d: *p* < 0.01. Values in parentheses are percentages (%)

In SACs smaller than 10 mm, there were no cases with submucosal invasion; however, in SACs larger than 20 mm, the incidence of T1 carcinoma in Group A (71%) was significantly higher than that in Group B (13%) (*p* < 0.05) **(**Table 5 **)**. Serrated adenocarcinoma characteristics listed according to their serrated adenoma are shown in Table 6.

**Table 5:** Incidence of submucosal invasion in serrated adenocarcinoma according to size and epithelial serration

Size (mm)	Epithelial serration		*p*-value
	Present (*n* = 17)	Absent (*n* = 23)	
<10	0% (0/0)	0% (0/1)	N.S.
11–20	67% (4/6)	17% (1/6)	N.S.
>20	73% (8/11)	13% (2/16)	<0.05
Total	70% (12/17)	13% (3/23)	<0.05

*N.S.* not significant

**Table 6:** Characteristics of serrated adenocarcinomas according to the serrated adenoma type

Variables	TSA (*n* = 27)	SSA/P (*n* = 5)	Unclassified (*n* = 5)	Non-serrated adenoma (*n* = 3)
Sex (male/female)	16/11	3/2	3/2	1/2
Average age (years)	69.1	69.0	68.6	73.3
Location (proximal colon/distal colon/rectum)	7/1/19	5/0/0	2/1/2	2/0/1
Average size (mm)	42.6^a^	17.2^b^	34.0	18.3^c^
Invasion depth (Tis/T1)	24/3^d^	1/4^e^	0/5^f^	0/3^g^
Epithelial serration (+/−)	6/21	3/2	5/0	3/0

*TSA* traditional serrated adenoma, *SSA/P* serrated adenoma/polyp

a vs. b and c, d vs. e, f, and g: *p* < 0.01

TSA-type tumors were significantly larger than SSA and non-serrated adenoma type lesions. The TSA type SACs were mainly located in the rectum. The SSA type SACs were located in the proximal colon in all cases. The incidences of submucosal invasion in SSA type (80%), unclassified type (100%) and non-serrated adenoma type (100%) lesions were significantly greater than those of the TSA type (11%) **(**Table 6 **)**. T1 SACs were observed in 15 patients (12 in Group A and 3 in Group B). For T1 SACs, vessel invasion was observed in 4 Group A patients. Among surgical patients, lymph node metastasis was observed in 2 patients in Group A; none of the patients in group B had vessel invasion or lymph node metastasis **(**Table 7 **)**.

**Table 7:** Fifteen cases of T1 serrated adenocarcinoma

Case No.	Sex	Age (decade years)	Location	Size (mm)	Epithelial serration	Serrated adenoma	SM invasion depth (μm)	Budding grade	Vessel invasion	Lymph node metastasis
1.	Female	50s	C/C	30	+	+	200	1	ly0, v0	–
2.	Female	70s	Rb	40	+	+	1000	2	ly0, v0	–
3.	Male	70s	Rb	40	+	+	1100	3	ly0, v0	–
4.	Male	70s	D/C	30	+	+	1400	2	ly0, v0	–
5.	Male	60s	C/C	10	+	+	1500	1	ly0, v0	–
6.	Male	60s	A/C	30	+	+	1500	1	ly3, v1	+
7.	Female	80s	C/C	20	+	+	1500	1	ly0, v0	–
8.	Male	70s	A/C	15	+	–	1900	3	ly1, v1	–
9.	Female	80s	C/C	10	+	–	2000	3	ly0, v0	–
10.	Female	60s	Ra	30	+	–	2500	3	ly3, v0	+
11.	Female	80s	Rb	50	+	+	2600	1	ly1, v0	–
12.	Male	60s	T/C	30	+	+	3000	1	ly0, v0	–
13.	Male	60s	A/C	20	–	+	170	1	ly0, v0	–
14.	Male	50s	Rb	90	–	+	3000	3	ly0, v0	–
15.	Male	50s	Ra	40	–	+	5200	2	ly0, v0	–

*C/C* Cecum, *A/C* Ascending colon, *T/C* Transverse colon, *D/C* Descending colon, *Ra* Rectum above the peritoneal reflection, *Rb* Rectum below the peritoneal reflection, *SM* submucosal

## Discussion

To our knowledge, this is the first study of the clinicopathologic and endoscopic features of early-stage SAC. The discovery of SAC was anticipated by the recognition of high-grade dysplasia or Tis carcinoma in TSA in several studies [23–28]. The ages and sexes of our SAC patients were consistent with those reported previously [29, 30]. SACs were observed predominantly in the proximal colon and the rectum. Those in the proximal colon appear to be more related to SSAs; however, most distal SACs likely originate from TSAs since SSAs were located predominantly in the right colon whereas TSAs were located in the left colon [24, 29, 30]. The diagnosis of SACs with poorly differentiated carcinoma or without an adjacent serrated adenoma is difficult in advanced stages. In SACs with mucinous components, the serrated growth pattern is well presented; however, displacement may compress the epithelium so that the serrated projections are not apparent, or an abundance of mucus in combination with poor differentiation may render the serrated pattern unrecognizable. We were able to detect minute changes in serrated morphology and in the serrated components close to the carcinoma.

Our study also showed that group A tumors exhibited more aggressive behavior and malignant potential, even though the average size of SACs with epithelial serration was smaller than that of SACs with non-epithelial serration. Of the T1 carcinomas in Group A, 67% were 11–20 mm in size. Molecularly distinct subtypes of colorectal carcinomas, including those that develop from serrated precursor lesions, are considered to have poor prognoses [31]. Carcinomas of the serrated pathway without MSI are aggressive; SACs with epithelial serration tend to exhibit submucosal invasion, even during early stages. The incidences of vessel invasion in SACs with epithelial serration were significantly higher than those in SACs with non-epithelial serration. Therefore, epithelial serration appears to be a predictor of aggressive carcinoma.

Endoscopic findings revealed that the incidence of type V pit pattern in SACs with epithelial serration was significantly higher than that in SACs with non-epithelial serration. Recently, the JNET established a universal NBI magnifying endoscopic classification system for colorectal tumors [15, 16], according to which submucosal deep invasive colorectal carcinomas were classified as type 3. The incidences of JNET type 3 lesions in SACs with epithelial serration were significantly higher than those in SACs with non-epithelial serration. NBI magnification is also useful to predict the depths for SACs. The latest WHO classification categorizes SACs into 3 groups: HP, SSA/polyps (SSA/P), and TSA; the endoscopic features of conventional SSA/P have a pale color similar to HPs. When observed with crystal violet staining under magnification, the orifices are observed to be widely open, and are referred to as type II-open pit [32]. SSAs are also classified as type 1 according to the JNET classification when observed with NBI magnification [15, 16]. VMVs are defined as vessels thicker than meshed capillary ducts that have meander-like flow resembling varicose veins; this is distinct from the capillary pattern of the mucosal vascular network [33]. The endoscopic features of protruded-type TSAs include enhanced-reddish villous lesions [34]. As for macroscopic features, Sano et al. documented the pinecone-like appearance as characteristic of TSA [35]; when observed with crystal violet staining under magnification, the type IV pit pattern is often present, and is associated with the type II pit pattern at the base [36]. When observed with magnifying NBI, TSAs were consequently classified as type 2A according to the JNET guidelines [15, 16]; therefore, it is possible to distinguish between TSA and SSA endoscopically using magnification. There were no significant differences in pinecone-like findings and VMVs between Group A and Group B, even though Group A tended to exhibit more of the latter while Group B had more of the former. Moreover, distinct areas or transition points in which the pit pattern changes from type II to type III or IV suggest the development of a dysplasic area [37]. Therefore, determining the origins of SACs remains challenging.

There were some limitations in this study, including its retrospective, single-center nature and the relatively small number of SACs owing to the rarity of such lesions. A large, multicenter prospective trial is required for further validation of our findings.

## Conclusions

We found that epithelial serration in the cancerous area of early-stage SACs and a non-TSA serrated adenoma background are independent predictors of aggressive behavior. Our results may be helpful for determining indications of endoscopic resection in patients with serrated lesions. Further research ought to elucidate the molecular or genetic mechanisms behind the aggressive behavior of early-stage SACs.

## Abbreviations

HP: Hyperplastic polyp
JNET: Japan NBI Expert Team
MSI: Microsatellite instability
NBI: Narrow-band imaging
SAC: Serrated adenocarcinoma
SSA: Sessile serrated adenoma
SSA/P: SSA/polyps
TSA: Traditional serrated adenoma
VMV: Varicose microvascular vessels
WHO: World Health Organization
